# Analyzing the non-linear relationship between fasting blood glucose levels and Gensini score in patients with STEMI

**DOI:** 10.3389/fcvm.2024.1427567

**Published:** 2024-12-19

**Authors:** Han Li, Quanzhi Lin, Zhiyuan Jiang, Guoqiang Zhong

**Affiliations:** ^1^ Department of Cardiology, The First Affiliated Hospital of Guangxi Medical University, Nanning, Guangxi, China; ^2^ Department of Cardiology, The First Affiliated Hospital of Guangxi University of Science and Technology, Liuzhou, Guangxi, China

**Keywords:** acute myocardial infarction, Gensini score, fasting blood glucose, ST-elevation myocardial infarction, diabetes mellitus

## Abstract

**Background:**

Acute myocardial infarction (AMI), particularly ST-segment elevation myocardial infarction (STEMI), significantly impacts global health, exacerbated by risk factors such as diabetes mellitus (DM). While the Gensini score effectively quantifies coronary artery lesions, its correlation with fasting blood glucose (FBG) levels, particularly in a non-linear fashion, has not been thoroughly explored in STEMI patients.

**Methods:**

This study analyzed data from 464 STEMI patients treated with percutaneous coronary intervention at the First People's Hospital of Taizhou City, Zhejiang Province, China, from January 2010 to October 2014. We stratified patients into three FBG tertiles and utilized multiple statistical analyses, including least absolute shrinkage and selection operator (LASSO) regression and curve fitting, to examine the potential U-shaped relationship between FBG levels and Gensini scores.

**Results:**

Our analysis revealed significant differences in Gensini scores across FBG tertiles, with both hypoglycemic and hyperglycemic extremes showing higher scores compared to the normoglycemic range. The curve fitting analysis confirmed a U-shaped relationship, suggesting a significant, non-linear association between FBG levels and coronary artery lesion severity, regardless of diabetes status.

**Conclusions:**

Our findings underscore the complexity of glycemic control in STEMI management and suggest that both hypo- and hyperglycemia are significant risk factors for severe coronary lesions as quantified by the Gensini score. This study highlights the importance of comprehensive FBG monitoring and management to improve outcomes for STEMI patients.

## Introduction

Acute myocardial infarction (AMI), caused by occlusion or severe narrowing of coronary arteries due to clot formation in atherosclerotic plaque (1), affects over 7 million people globally each year (2) and carries a high mortality rate (3). ST-segment elevation myocardial infarction (STEMI) is a particularly prevalent type of AMI and a leading cause of death and illness among major coronary events (4). Diabetes mellitus (DM) increases cardiovascular morbidity and mortality, especially in individuals with acute coronary syndrome (ACS), myocardial infarction (MI), or unstable angina pectoris (5). Poor glycemic control is typically associated with a worsening of coronary heart disease risk. Fasting hyperglycemia detected after a heart attack is linked to poorer outcomes (6). It can also help predict both future heart disease risk and the development of diabetes (7). The Gensini score reflects the severity of coronary artery lesions, and higher fasting blood sugar (FBG) levels are correlated with a higher Gensini score (8, 9). Over the years, studies have shown a significant positive correlation between fasting glucose and Gensini scores, even in people without diabetes (10–13). Previous research has suggested a linear relationship between blood sugar and Gensini score. However, these studies only considered the influence of blood sugar, not other factors like uric acid, creatinine, and age. Additionally, previous analyses used a single cutoff to define high-risk hyperglycemia (14). Both hypoglycemia and hyperglycemia are well-established risk factors for AMI. Therefore, our study used three FBG cutoff values and adjusted for other variables to evaluate their association with Gensini scores. We aimed to investigate the potential U-shaped relationship between FBG concentration and Gensini score analysis in STEMI patients. By understanding this relationship, we hope to improve our understanding of STEMI pathophysiology and identify potential therapeutic targets to optimize patient management and improve outcomes.

## Methodology and materials overview

### Participant demographics and characteristics

All data for this study are publicly available on the Dryad Digital Repository (https://datadryad.org/stash/dataset/doi:10.5061/dryad.pf56m) without restrictions. The data emanated from a study titled “Predictive value of Apelin-12 in patients with STEMI with different renal function: a prospective observational study”. Between January 2010 and October 2014, 464 patients with symptoms of STEMI undergoing percutaneous coronary intervention (PCI) were enrolled at the First People's Hospital of Taizhou City, Zhejiang Province, China. These patients presented with sustained chest pain (>30 min), prolonged electrocardiographic changes (including ischemic ST-segment elevation in two or more contiguous leads), along significantly elevated serum cardiac enzyme and troponin concentrations. This study involved secondary analysis of previously collected data, making participant consent or approval from an ethics committee unnecessary. Patients were excluded if they had non-STEMI acute coronary syndrome, prior balloon angioplasty without stenting, severe vascular heart disease, cardiogenic shock, secondary hypertension, history of stroke within the past year or ongoing stroke with significant neurological impairment, prior rescue PCI procedure, documented ventricular fibrillation, planned conservative treatment without PCI, chronic hepatitis or cirrhosis, untreated severe heart block, life expectancy less than 12 months, endocrine disorders like thyroid or adrenal dysfunction, severe kidney failure requiring dialysis, recent major infection, connective tissue disease, malignant tumor, known allergies to medications used in the study (statins, heparin, aspirin, clopidogrel, contrast dye, or GPIIb/IIIa inhibitors), active bleeding in the digestive or urinary tract, major surgery or trauma within the past 6 weeks with incomplete medical records, or ongoing severe bleeding.

### Assessment methods

Coronary artery assessment was performed using coronary angiography on all participants. This procedure evaluated the degree of stenosis in the left main coronary artery, left anterior descending artery, left circumflex artery, and right coronary artery, along with imaging findings. A normal coronary artery had no narrowing or less than 50% stenosis. A single stenosis was identified if any of the arteries had 50% or more narrowing. In contrast, a multi-branch lesion was confirmed if two or three arteries had ≥50% stenosis. The American Heart Association's Gensini score was used to grade stenosis severity: 1 for ≤25%, 2 for 26%–50%, 4 for 51%–75%, 8 for 76%–90%, 16 for 91%–99%, and 32 for complete occlusion (15). Diabetes was defined as a pre-existing condition requiring medication or non-pharmacological interventions or new-onset diabetes diagnosed according to the American Diabetes Association criteria, which include a medical history of typical symptoms accompanied by elevated blood glucose levels [fasting plasma glucose ≥126 mg/dl during hospitalization or hemoglobin A1c≥6.5% (48 mmol/mol), or a random or 2-h postprandial blood glucose ≥200 mg/dl] (16). Hypertension was defined as repeated blood pressure readings ≥140/90 mmHg on at least two separate occasions and was presumed to be present in individuals taking antihypertensive medication. The Killip classification is a systematic method for assessing the severity and prognosis of acute AMI patients based on clinical signs at admission, including heart murmurs, lung congestion sounds (rales), edema, and low blood pressure. It has four levels: Killip I: No signs of heart failure; Killip II: Lung congestion sounds or murmurs without heart failure; Killip III: Lung congestion sounds and signs of heart failure; Killip IV: Cardiogenic shock (severe heart failure) (17).

### Statistical analysis

The study participants were stratified into three groups based on their blood glucose levels: tertile 1 (2.69–6.15 mmol/L), tertile 2 (6.19–8.61 mmol/L), and tertile 3 (8.64–14.81 mmol/L). Subsequently, the differences in Gensini scores among these groups were assessed. To assess data normality, the Shapiro-Wilk test was used. Normally distributed continuous data were expressed as mean ± standard deviation and analyzed using ANOVA with LSD *post hoc* test for intergroup comparisons. Non-normally distributed data were presented as median (minimum-maximum), and analyzed using the Kruskal-Wallis test for group differences. Categorical variables were presented as counts and percentages (%). In univariate analysis, we investigated the relationship between various factors (independent variables) and the Gensini score (dependent variable). To further explore the association between blood glucose and Gensini score, we employed least absolute shrinkage and selection operator (LASSO) regression analysis for covariate selection. We then performed multivariate regression analysis with the Gensini score as the dependent variable, glucose as the independent variable, and selected variables as covariates, aiming to adjust for confounding factors and assess the true association between glucose and the Gensini score. Recognizing that a linear relationship may not exist between blood glucose and the Gensini score, curve fitting was employed to observe the trend in blood glucose and the Gensini score. Statistical analysis was performed using R version 3.6.1 (https://www.r-project.org/), with a significance level set at *p* < 0.05 (two-sided) to denote statistical significance.

## Results

### Baseline patient characteristics in the study cohort

Table 1 presents a cohort of 464 subjects stratified into three groups based on fasting blood glucose levels. Tertile 1 had a mean level of 5.30 mmol/L (range: 2.69–6.15 mmol/L), tertile 2 had a mean of 7.11 mmol/L (range: 6.19–8.61 mmol/L), and tertile 3 had a mean of 10.70 mmol/L (range: 8.64–14.81 mmol/L). Baseline characteristics, including anatomical, hematological, and demographic parameters, were compared across these groups. Significant differences were observed in total cholesterol and Gensini score (*p* < 0.001). Detailed baseline characteristics are provided in Table 2. The overall cohort had a mean age of 63 years (63.00 ± 11.92) and a mean fasting blood glucose level of 7.67 mmol/L (7.67 ± 2.53). There were 355 males and 109 females, with 314 subjects without diabetes and 150 diagnosed with diabetes. Patients presented with KILLIP grades of 1–2, and the main culprit vessels in coronary artery disease leading to myocardial infarction were predominantly one or three vessels. Univariate analysis revealed significant correlations (*p* < 0.05) between Gensini scores and various parameters, including hemoglobin, low-density lipoprotein, D-dimer, blood glucose, leukocyte count, systolic blood pressure, myocardial infarction history, creatinine, uric acid, total cholesterol, CTN, CKM, left ventricular end-diastolic diameter, and left atrial diameter. Notably, fasting blood glucose exhibited a positive correlation with the Gensini score (*r* = 2.0, *p* < 0.0007).

**Table 1:** Characteristics of the study participants stratified by fasting blood glucose tertiles.

Glucose tertile	Low	Middle	High	*P*-value
*N*	155	154	155	
Neutrophils, 10^9^/L	76.70 (51.00–95.80)	78.35 (40.60–98.30)	76.60 (43.90–94.60)	0.251
Hemoglobin, g/L	145.00 (97.00–187.00)	142.00 (88.00–179.00)	143.00 (108.00–172.00)	0.316
Platelet, 10^9^/L	219.00 (121.00–342.00)	232.00 (110.00–343.00)	240.00 (123.00–368.00)	0.593
Albumin, g/L	38.00 (28.80–45.30)	38.00 (18.10–46.80)	38.00 (30.20–45.70)	0.655
Triglycerides, mmol/L	0.92 (0.00–3.76)	0.95 (0.04–4.12)	1.10 (0.01–10.25)	0.431
Low density lipoprotein, mmol/L	3.00 (1.42–4.24)	3.00 (1.57–5.43)	3.00 (1.77–6.64)	0.656
Dimer, mg/L	0.82 (0.00–10.00)	0.90 (0.00–8.60)	1.14 (0.00–2.60)	0.042
Apelin, ng/ml	0.78 (0.06–2.36)	0.80 (0.25–1.87)	0.73 (0.18–2.31)	0.503
Urea nitrogen, mmol/L	6.70 (2.70–11.20)	6.70 (2.30–11.50)	6.90 (2.40–11.80)	0.513
Glucose, mmol/L	5.30 (2.69–6.15)	7.11 (6.19–8.61)	10.70 (8.64–14.81)	<0.001
White blood cells, 10^9^/L	9.96 (2.96–22.26)	9.46 (2.16–21.34)	9.96 (3.96–16.64)	0.378
Systolic blood pressure, mm/Hg	133.00 (79.00–181.00)	127.00 (76.00–177.00)	134.00 (87.00–196.00)	0.148
Heart rate, n/min	75.00 (32.00–129.00)	75.00 (43.00–143.00)	77.00 (44.00–135.00)	0.378
Age, *N*	64.00 (29.60–90.70)	63.00 (28.00–87.00)	61.30 (25.70–86.90)	0.144
Creatinine, mmol/L	75.00 (43.90–208.50)	75.30 (35.90–116.70)	74.00 (35.70–386.80)	0.877
Uric acid, mmol/L	340.00 (162.50–520.00)	340.00 (171.60–548.10)	330.00 (163.10–533.10)	0.383
Total cholesterol, mmol/L	5.66 (0.66–8.03)	5.48 (1.43–8.89)	5.94 (3.77–11.85)	<0.001
High density lipoprotein, mmol/L	1.21 ± 0.28	1.20 ± 0.27	1.19 ± 0.26	0.89
Ctni, ng/ml	16.40 (0.02–41.10)	11.95 (0.02–43.20)	12.60 (0.02–43.13)	0.355
Ckmb, ng/ml	106.00 (10.00–451.00)	102.50 (9.00–357.00)	113.00 (16.00–296.00)	0.518
Gensini score	67.00 (18.00–133.00)	64.50 (18.00–128.00)	88.00 (20.00–127.00)	<0.001
Left ventricular end diastolic diameter, mm	50.00 (38.00–70.00)	52.00 (37.00–64.00)	49.00 (38.00–64.00)	0.172
Left atrial diameter, mm	38.00 (24.00–50.00)	38.00 (25.00–51.00)	38.00 (24.00–48.00)	0.988
Stent number, *n*	1.00 (1.00–3.00)	1.00 (1.00–3.00)	1.00 (1.00–3.00)	0.743
Sex				
1 = Male	119 (76.77%)	113 (73.38%)	123 (79.35%)	
2 = Female	36 (23.23%)	41 (26.62%)	32 (20.65%)	
Htn				0.067
0	59 (38.06%)	62 (40.26%)	78 (50.32%)	
1	96 (61.94%)	92 (59.74%)	77 (49.68%)	
Dm, mmol/L				0.047
0	116 (74.84%)	102 (66.23%)	96 (61.94%)	
1	39 (25.16%)	52 (33.77%)	59 (38.06%)	
Pathological q wave				0.778
1	71 (45.81%)	75 (48.70%)	77 (49.68%)	
2	84 (54.19%)	79 (51.30%)	78 (50.32%)	
Culprit vessels, *n*				0.236
1	71 (45.81%)	76 (49.35%)	86 (55.48%)	
2	29 (18.71%)	19 (12.34%)	24 (15.48%)	
3	55 (35.48%)	59 (38.31%)	45 (29.03%)	
Myocardiol infarction history				0.662
1	16 (10.32%)	21 (13.64%)	18 (11.61%)	
2	139 (89.68%)	133 (86.36%)	137 (88.39%)	
Anterior wall myocardial infarction				0.226
1	69 (44.52%)	78 (50.65%)	84 (54.19%)	
2	86 (55.48%)	76 (49.35%)	71 (45.81%)	
Killip grade				0.46
1	123 (79.35%)	114 (74.03%)	115 (74.19%)	
2	32 (20.65%)	40 (25.97%)	40 (25.81%)	

CK-MB, creatine kinase MB; cTnI, cardiac troponin I; DM, diabetes mellitus.

**Table 2:** Univariate analysis related to the Gensini score.

	Statistics	Gensini score
Neutrophils	75.70 ± 11.50	−0.14 (−0.39, 0.12) 0.2858
Hemoglobin	143.78 ± 17.15	0.19 (0.02, 0.36) 0.0297
Platelet	231.98 ± 56.13	0.03 (−0.02, 0.08) 0.2430
Albumin	37.94 ± 3.83	−0.62 (−1.39, 0.14) 0.1114
Triglycerides	1.11 ± 0.84	1.29 (−2.22, 4.79) 0.4718
Low density lipoprotein	3.05 ± 0.72	4.37 (0.34, 8.41) 0.0342
Dimer	1.06 ± 1.02	4.53 (1.67, 7.39) 0.0020
Apelin	0.83 ± 0.34	−2.95 (−11.71, 5.80) 0.5088
Urea nitrogen	6.74 ± 2.07	0.63 (−0.78, 2.05) 0.3806
Glucose	7.67 ± 2.53	2.00 (0.85, 3.15) 0.0007
White blood cells	10.05 ± 3.66	0.85 (0.05, 1.65) 0.0380
Systolic blood pressure	131.78 ± 27.15	0.12 (0.01, 0.23) 0.0286
Heart rate	76.95 ± 17.13	−0.02 (−0.19, 0.15) 0.8342
Age	63.00 ± 11.92	−0.23 (−0.48, 0.01) 0.0643
Sex		
1（1 = Male)	355 (76.51%)	Reference
2（Female)	109 (23.49%)	−1.95 (−8.87, 4.97) 0.5805
HTN		
0	199 (42.89%)	Reference
1	265 (57.11%)	0.31 (−5.62, 6.24) 0.9181
Dm		
0	314 (67.67%)	Reference
1	150 (32.33%)	−1.39 (−7.66, 4.89) 0.6648
Culprit vessels		
1	233 (50.22%)	Reference
2	72 (15.52%)	5.79 (−2.72, 14.31) 0.1831
3	159 (34.27%)	0.34 (−6.16, 6.84) 0.9178
Pathological Q wave		
1	223 (48.06%)	Reference
2	241 (51.94%)	−2.45 (−8.32, 3.43) 0.4147
Myocardiol infarction history		
1	55 (11.85%)	Reference
2	409 (88.15%)	9.58 (0.54, 18.62) 0.0384
Anterior wall myocardial infarction		
1	231 (49.78%)	Reference
2	233 (50.22%)	−1.04 (−6.91, 4.83) 0.7282
Killip grade		
1	352 (75.86%)	Reference
2	112 (24.14%)	3.33 (−3.53, 10.18) 0.3417
Stent number		
1	302 (65.09%)	Reference
2	148 (31.90%)	−1.39 (−7.72, 4.95) 0.6679
3	14 (3.02%)	12.19 (−5.07, 29.45) 0.1670
Creatinine	74.62 ± 22.71	−0.25 (−0.38, −0.13) 0.0001
Uric acid	337.18 ± 73.69	−0.05 (−0.09, −0.01) 0.0106
Total cholesterol	5.64 ± 1.13	3.55 (0.97, 6.12) 0.0072
High density lipoprotein	1.20 ± 0.27	−1.49 (−12.28, 9.30) 0.7872
Ctni	16.51 ± 12.85	0.21 (−0.01, 0.44) 0.0662
Ckmb	127.29 ± 89.58	0.04 (0.00, 0.07) 0.0333
Left ventricular end diastolic diameter	50.45 ± 6.27	0.62 (0.15, 1.08) 0.0095
Left atrial diameter	37.42 ± 5.66	0.71 (0.20, 1.23) 0.0069

HTN, 1 = hypertension, 0 = not hypertension; DM, 1 = diabetes mellitus, 0 = not diabetes mellitus; culprit vessels, 1 = left anterior descending, 2 = left circumflex coronary artery, 3 = right coronary artery; pathological Q wave, 1 = yes, 2 = no; myocardial infarction history, 1 = yes, 2 = no; anterior wall myocardial infarction, 1 = yes, 2 = no; CK-MB, creatine kinase MB; cTnI, cardiac troponin I.

### Principal findings

**Figure 1:**
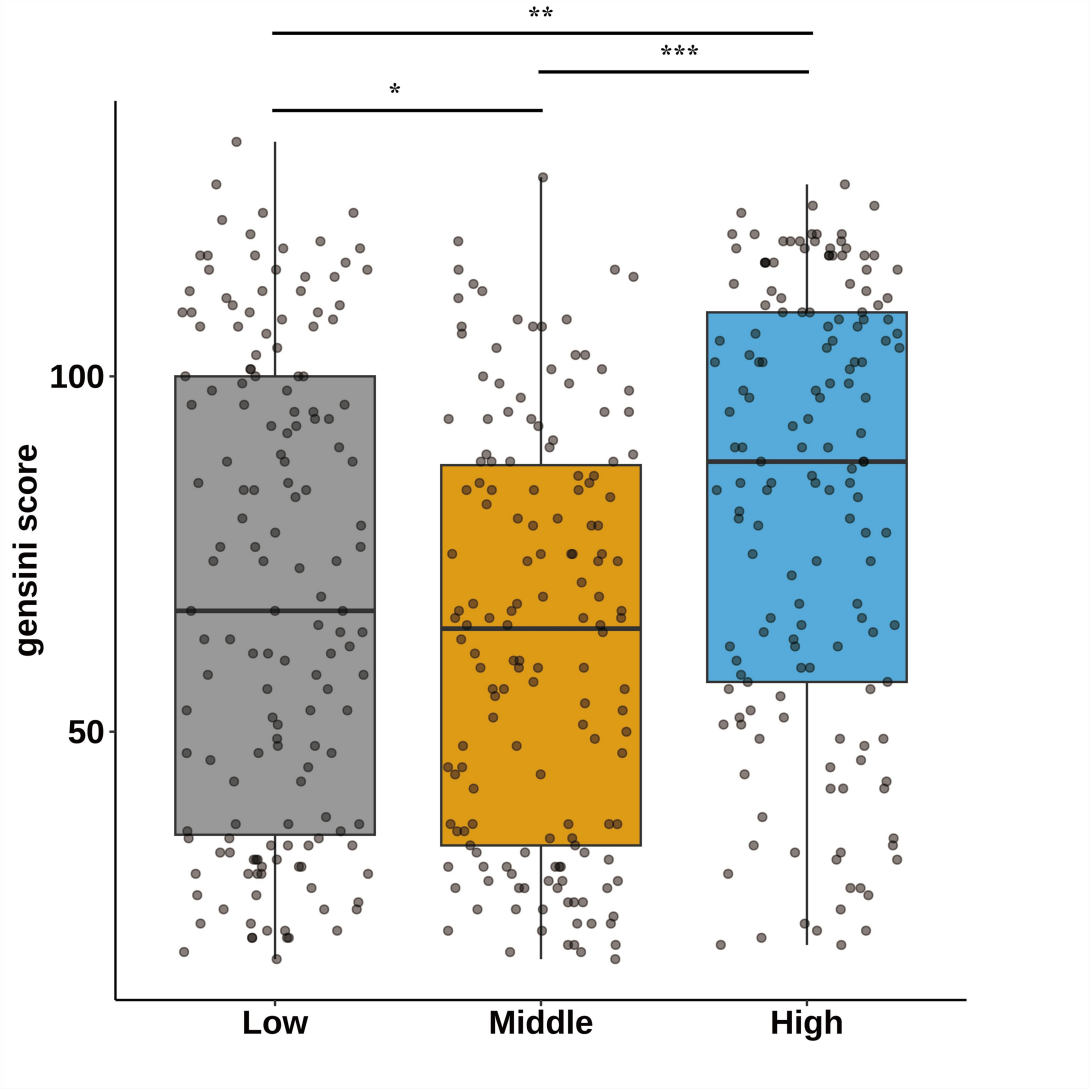
Characteristics of the study participants stratified by fasting blood glucose tertiles; *p < 0.05,*p < 0.01,*p < 0.001. Fasting blood glucose was divided into hypoglycemic group 5.30 mmol/L (2.69–6.15), moderate glycemic group 7.11 mmol/L (6.19–8.61), and hyperglycemic group 10.70 mmol/L (8.64–14.81).

**Figure 2:**
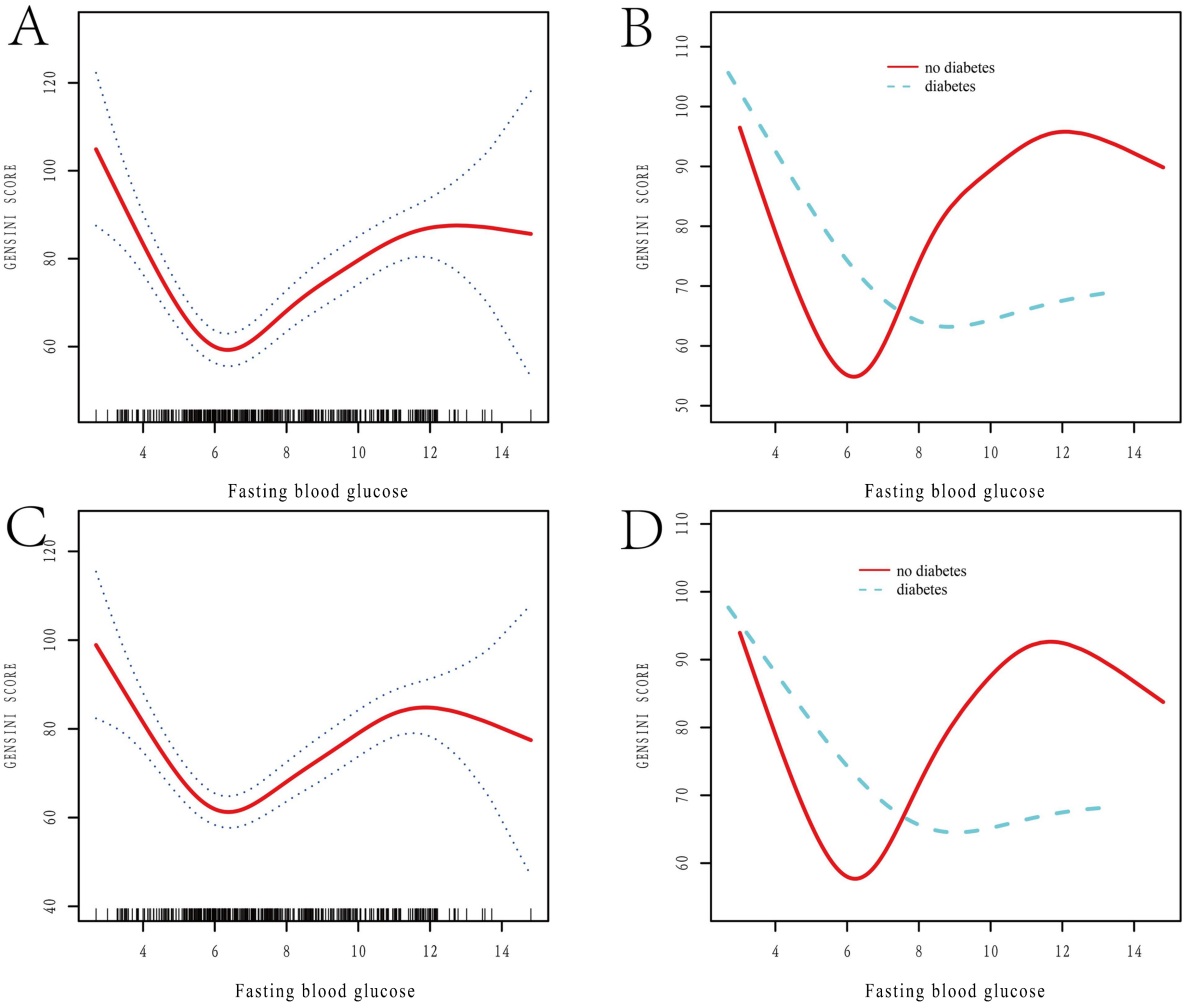
Restricted spline curve analysis of the relationship between FBG levels and Gensini score. **(A,B)** Unadjusted Variable Curves Fitted to Model-Constrained Spline Curves; **(A)** the relationship between FBG levels and Gensini score; **(B)** the relationship between FBG levels and Gensini score in diabetic and non-diabetic patients; FBG: fasting blood glucose. **(C,D)** Curve Fitting After Adjustment for Variables; Adjusted variables include neutrophils, hemoglobin, platelets, albumin, low-density lipoprotein, D-dimer, urea nitrogen, glucose, white blood cells, systolic blood pressure, heart rate, age, DM, culprit vessels, myocardial infarction history, Killip grade, stent number, creatinine, uric acid, total cholesterol, CTNI, CKMB, left ventricular end-diastolic diameter, and left atrial diameter. **(C)** The relationship between adjusted FBG levels and Gensini score, after controlling for variables; **(D)** the relationship between adjusted FBG levels and Gensini score in diabetic and non-diabetic patients, after controlling for variables. FBG, fasting blood glucose.

Figure 1 categorizes fasting blood glucose into three groups: hypoglycemic (5.30 mmol/L, range: 2.69–6.15 mmol/L), moderate glycemic (7.11 mmol/L, range: 6.19–8.61 mmol/L), and hyperglycemic (10.70 mmol/L, range: 8.64–14.81 mmol/L), with statistically significant differences across these subgroups (*p* < 0.05). Table 3 shows the Gensini scores without adjusting for other variables. Scores in the moderate glycemic and hyperglycemic groups exhibited significant clinical significance (moderate glycemic group: 95% CI −140,162–0.213, *p* = 0.044; hyperglycemic group: 95% CI 5.295–19.221, *p* = 0.0006). To identify relevant variables for further analysis (Model 2), the LASSO regression was employed. The screening criterion used was lambda = lambdam.min:0.8132 (−0.2068). This identified the following variables for inclusion: neutrophils, hemoglobin, platelets, albumin, low-density lipoprotein, D-dimer, urea nitrogen, glucose, white blood cells, systolic blood pressure, heart rate, age, DM diagnosis, culprit vessels, myocardial infarction history, Killip grade, number of stents, creatinine, uric acid, total cholesterol, cTnI, CKMB, left ventricular end-diastolic diameter, and left atrial diameter. Following adjustment for these confounding factors, only the hyperglycemic group remained significantly associated with the Gensini score (95% CI: −16.95 to −2.71, *p* = 0.015). Finally, curve fitting analysis (Figure 2) revealed a U-shaped, non-linear relationship between fasting glucose and the Gensini score in patients, regardless of their diabetes diagnosis (Figure 2).

**Table 3:** Curve fitting model of fasting blood glucose and Gensini scores.

	Unadjusted model		Model 1	
	Β (95%CI)	*P* Value	Β (95%CI)	*P* Value
Glucose	2.003 (0.854, 3.151)	0.0007	1.71 (0.60, 2.83)	0.0027
Glucose tertile				
Low	Reference		Reference	
Middle	−7.188 (−14.162, −0.213)	0.044	−5.71 (−12.35, 0.94)	0.0929
High	12.258 (5.295, 19.221)	0.0006	10.97 (4.23, 17.72)	0.0023

Model 1, Unadjusted model. Model 2, Adjusted for all covariates: Neutrophils, hemoglobin, platelet, albumin, low-density lipoprotein, d dimer, urea nitrogen, glucose, white blood cells, systolic blood pressure, heart rate, age, DM, culprit vessels, myocardial infarction history, Killip grade, stent number, creatinine, uric acid, total cholesterol, cTnI, CKMB, left ventricular end-diastolic diameter and left atrial diameter.

## Discussion

MI is a common cause of death among diabetic patients (18). Studies have documented a U-shaped or J-shaped relationship between various blood glucose measures (fasting, admission, mean hospitalized) and in-hospital mortality in AMI patients (14, 19–21). However, some studies have reported contrasting findings. Park et al. found a J-shaped association between FBG and atherosclerotic cardiovascular disease (22). Several studies have explored the correlation between fasting blood glucose and the Gensini score, a measure of coronary artery disease severity. Yuhan Qin et al. observed a positive correlation (*r* = 0.171, *p* < 0.001) in 958 AMI patients (10). In a study by Tong Zhao et al., which included 64 patients with chest discomfort undergoing coronary angiography, FBG was positively correlated with Gensini score in both diabetic (*r* = 0.312, *p* < 0.012) and non-diabetic patients (*r* = 0.387, *p* < 0.010) (11). Besides, Jingjing Jiang et al. analyzed data from 1,852 patients who underwent coronary angiography. Their findings revealed a positive correlation between FBG and Gensini score (*r* = 0.09, *p* < 0.01). Regression analysis further demonstrated an independent correlation between FBG and Gensini score (12). In another study involving 85 patients with acute coronary syndrome who underwent emergency coronary angiography and received stent implantation when necessary, FBG was found to be positively correlated with the Gensini score (*r* = 0.568, *p* < 0.000) (8). Finally, a significant positive correlation was observed between FBG and Gensini score in 906 patients who underwent coronary angiography and had no history of diabetes (*r* = 0.172; *p* = 0.011) (13). Previous studies utilized a single truncation method to identify high-risk hyperglycemia (14). However, this approach overlooks the potential detrimental effects of hypoglycemia on coronary artery disease severity. To address this limitation, our study categorized participants into three fasting blood glucose groups: hypoglycemic (5.30 mmol/L, range: 2.69–6.15 mmol/L), moderate glycemic (7.11 mmol/L, range: 6.19–8.61 mmol/L), and hyperglycemic (10.70 mmol/L, range: 8.64–14.81 mmol/L). We further adjusted for a comprehensive set of covariates, including demographic factors (age), medical history (diabetes mellitus, myocardial infarction), laboratory values (neutrophils, hemoglobin, platelets, albumin, etc.), and measures of coronary artery disease severity (culprit vessels, Killip grade, stent number, etc.) to evaluate their association with Gensini scores. This approach revealed a U-shaped, rather than linear, relationship between fasting blood glucose and Gensini score in our population-based study.

Both hypoglycemia and hyperglycemia can exacerbate the severity of coronary artery lesions. To gain a deeper understanding of the mechanisms underlying this relationship, we have proposed several hypotheses, attempting to explain the potential mechanisms from a pathophysiological perspective. It is now understood that hyperglycemia damages the microvascular endothelial barrier and promotes atherosclerosis (23). A key mechanism involves endothelial dysfunction through the protein kinase C (PKC) pathway. This dysfunction is caused by increased non-enzymatic glycosylation, oxidative stress, and decreased endothelial insulin action (24). High glucose concentrations further injure blood vessels by activating nuclear factor-κB (NF-κB) and inducing overexpression of genes in endothelial cells, macrophages, and smooth muscle cells (25). Prolonged exposure to high glucose leads to the formation of advanced glycation end products (AGEs), which generate reactive oxygen species (ROS). Glucose oxidation by ROS damages tissues in the artery walls, contributing to diabetic macrovascular complications (26). Diabetic patients exhibit increased ROS production in cardiovascular cells, and activation of intercellular adhesion molecule-1 (ICAM-1) accelerates atherosclerosis progression (27, 28). Hyperglycemia also potentiates thrombosis, increasing the risk of coronary events (29, 30), mediated by augmenting platelet activation and aggregation, impairing fibrinolysis, and enhancing thrombin generation, ultimately leading to intravascular thrombus formation. Hyperglycemia disrupts the coagulation cascade, leading to increased fibrin deposition and stabilization within coronary arteries. This worsens luminal obstruction and can precipitate myocardial infarction (31). It also exacerbates myocardial oxygen demand and impairs coronary microvascular function, further promoting ischemic injury. The increased oxygen demand results from hyperglycemia-induced insulin resistance and impaired myocardial glucose utilization (32, 33). Impaired microvascular function by hyperglycemia compromises myocardial perfusion and worsens ischemic injury. Collectively, these mechanisms mediated by hyperglycemia contribute to elevated Gensini scores, reflecting the increased severity of coronary artery disease. These mechanisms play a critical role in the formation and progression of coronary artery blockages, constituting a crucial point on one end of the U-shaped relationship curve.

Both hypoglycemia and hyperglycemia are linked to an increased risk of cardiovascular disease. Severe hypoglycemia can directly impact outcomes and mortality (34), with studies showing a higher incidence of stent thrombosis and artery occlusions following STEMI (35, 36). Hypoglycemia contributes to cardiovascular disease through various mechanisms, including vasoconstriction, activation of white blood cells, imbalances in the autonomic nervous system, and the release of inflammatory chemicals called cytokines (37). Research has shown that hypoglycemia increases levels of hormones like glucagon, cortisol, and growth hormone (38), which can elevate heart contractility and oxygen demand, potentially leading to adverse cardiovascular effects (35). Systematic reviews and meta-analyses encompassing over 325,000 hospitalized patients indicate that individuals experiencing hypoglycemia face an elevated risk of subsequent adverse cardiovascular events (39). These analyses consistently demonstrate a significant correlation between hypoglycemia and cardiovascular events across various patient populations, including critically ill and non-critically ill patients, diabetics and non-diabetics, and those with mild to severe hypoglycemia. The association remained consistent in both short-term and long-term follow-up studies (40). Hypoglycemia affects the severity of coronary artery lesions through these mechanisms, accounting for the other end of the U-shaped curve observed in our study.

FBG stands out as a superior prognostic indicator for mortality and fatal heart failure compared to admission blood glucose. Studies have shown that FBG is superior to admission blood glucose (ABG) in predicting 30-day mortality in patients with AMI without diabetes (41). Additionally, FBG is a better predictor of long-term mortality. Non-diabetic AMI patients with elevated ABG (>110 mg/dl or 6.11 mmol/L) have a nearly fourfold higher mortality risk compared to those with normal blood sugar levels (42). Interestingly, elevated ABG does not increase mortality risk if FBG is normal. Conversely, elevated FBG upon admission triples mortality rates, even if ABG is normal (43). These findings suggest that both high and low blood glucose levels have detrimental effects on the cardiovascular system, increasing the risk of events. Our study investigated patients with STEMI and observed a U-shaped correlation between FBG and Gensini score. This relationship held true for both diabetic and non-diabetic patients, highlighting the negative impacts of both hyperglycemia and hypoglycemia on the cardiovascular system. These findings suggest an optimal range for blood glucose control that may minimize the severity of coronary artery disease. In patients with low FBG levels, associated pathophysiological changes may lead to vascular instability, while high FBG levels could accelerate the progression of atherosclerosis. Therefore, maintaining FBG within an ideal range could be crucial for reducing the risk of severe coronary lesions and improving clinical outcomes. In future research, stem cell therapy could be explored as a potential approach to mitigate coronary artery lesions caused by glycemic abnormalities in STEMI patients. Stem cells not only have the potential to differentiate into cardiomyocytes and endothelial cells, but they also secrete anti-inflammatory and growth factors that may enhance microcirculation and facilitate myocardial repair. The observed U-shaped relationship between hyperglycemia and coronary lesion severity suggests that glycemic fluctuations play a crucial role in STEMI pathogenesis. Stem cell therapy may help reduce lesion risk by improving local microcirculation and alleviating inflammation and oxidative stress induced by hyperglycemia. Moreover, the combined application of stem cell therapy and glycemic management strategies represents a promising research avenue, offering new insights and therapeutic options for the treatment of STEMI patients. Despite its contributions, our study has limitations. First, we focused only on STEMI patients, leaving uncertainties about the generalizability of the FBG-Gensini score relationship to other types of myocardial infarction. Second, our analysis was limited to a specific blood glucose range (2.69–14.81 mmol/L). The relationship between Gensini score and blood glucose levels outside this range remains unexplored. Finally, our study's relatively small sample size may affect the strength of our findings. Further validation in larger cohorts is warranted.

## Conclusion

Our study in STEMI patients reveals a U-shaped correlation between FBG concentration and Gensini score, underlining the negative effects of both hyperglycemia and hypoglycemia on coronary artery disease severity. Understanding this relationship can inform the development of targeted therapies to improve patient outcomes.

## Data Availability

Publicly available datasets were analyzed in this study. This data can be found here: https://datadryad.org/stash/dataset/doi:10.5061/dryad.pf56m.
